# DEPRESSIVE SYMPTOMS: HAMPERING MAINTENANCE OF COGNITIVE TRAINING GAINS

**DOI:** 10.1093/geroni/igz038.1619

**Published:** 2019-11-08

**Authors:** Cynthia Felix, Simo Du, Brad Taylor, George Rebok

**Affiliations:** 1 Johns Hopkins Bloomberg School of Public Health, Baltimore, Maryland, United States; 2 University of Florida, Gainesville, Florida, United States

## Abstract

To study the impact of depressive symptoms on the effectiveness of cognitive training interventions, the ACTIVE 10-year follow up data was analyzed for cognition and difficulty in everyday functioning outcomes. Because depression is treatable, this topic is of high public health significance. Analyses showed that females had greater prevalence of depressive symptoms. Reasoning, speed of processing and daily function change scores were significantly worse in the depressive symptom group, while memory change score was non-significantly worse, showing poor proximal and downstream primary (functional) outcomes. Depressive symptoms may lead to lower retention of cognitive training benefits.

